# Identification of Cellular Proteins that Interact with Human Cytomegalovirus Immediate-Early Protein 1 by Protein Array Assay

**DOI:** 10.3390/v6010089

**Published:** 2013-12-31

**Authors:** Francisco Puerta Martínez, Qiyi Tang

**Affiliations:** Department of Microbiology/RCMI Program, Ponce School of Medicine and Health Sciences, Ponce, PR 00716, USA

**Keywords:** human cytomegalovirus (HCMV), major immediate-early (MIE), IE1, protein-protein interaction, protein array

## Abstract

Human cytomegalovirus (HCMV) gene expression during infection is characterized as a sequential process including immediate-early (IE), early (E), and late (L)-stage gene expression. The most abundantly expressed gene at the IE stage of infection is the major IE (MIE) gene that produces IE1 and IE2. IE1 has been the focus of study because it is an important protein, not only for viral gene expression but also for viral replication. It is believed that IE1 plays important roles in viral gene regulation by interacting with cellular proteins. In the current study, we performed protein array assays and identified 83 cellular proteins that interact with IE1. Among them, seven are RNA-binding proteins that are important in RNA processing; more than half are nuclear proteins that are involved in gene regulations. Tumorigenesis-related proteins are also found to interact with IE1, implying that the role of IE1 in tumorigenesis might need to be reevaluated. Unexpectedly, cytoplasmic proteins, such as Golgi autoantigen and GGA1 (both related to the Golgi trafficking protein), are also found to be associated with IE1. We also employed a coimmunoprecipitation assay to test the interactions of IE1 and some of the proteins identified in the protein array assays and confirmed that the results from the protein array assays are reliable. Many of the proteins identified by the protein array assay have not been previously reported. Therefore, the functions of the IE1-protein interactions need to be further explored in the future.

## 1. Introduction

Human cytomegalovirus (HCMV) has been defined as one of the major infectious agents related to AIDS. HCMV infects large populations in general and causes serious diseases in immunocompromised individuals [1,2,3]. A sequential process of viral events is characterized for CMV infection in permissive host cells [3], including viral entry, immediate-early (IE) and early gene expression, DNA replication, late (L) gene expression, viral packaging and release. Major immediate-early (MIE) gene expression is one of the earliest events during CMV infection. MIE genes are the most abundantly expressed viral genes at the IE stage and give rise to several nuclear phosphoproteins that are critical for the next stage of gene expression and viral replication [4,5,6,7,8,9,10,11,12,13,14]. Hence, the HCMV MIE gene has been the focus of study. IE1 is believed to be the first *de novo* viral protein produced after HCMV infection and is essential for HCMV replication when HCMV is infected at a low multiplicity of infection (MOI). 

In general, IE1 has been considered as a gene activator. How IE1 activates gene expression is not fully understood. Several mechanisms that are involved in this activation (by IE1) have been described by different groups. First, IE1 interacts with nuclear corepressors such as PML, Daxx, and Sp100, thereby reducing their repressive effects on gene expression [15,16]. It was found by different groups independently that IE1 interacts with HDAC and inhibits HDAC’s activity [15,17]. Second, IE1 disperses ND10, a nuclear domain that contains many different nuclear suppressive proteins and responds to interferon stimulation [18,19,20,21]. In addition, innate cellular defense directed by interferons was disrupted by IE1 via inhibiting JAK-STAT signaling and by interacting with STAT1 [22,23,24]. The IE1 protein counteracts virus- or type I IFN-induced ISG activation via complex formation with STAT1 and STAT2 resulting in the reduced binding of ISGF3 to ISREs [22,23,24,25]. Last, IE1 is important for HCMV to arrest the cell cycle in the G1 phase that favors the infected HCMV for the cellular microenvironment. IE1 binds the Rb-related p107 protein and relieves its repression of E2F-responsive promoters [26]; IE1 also induces p53 accumulation through activating the p53 pathway by increasing the levels of p19^Arf^ and by inducing the phosphorylation of p53 at Ser15 [27], which might also relate to the HCMV-caused “G1 arrest” of infected cells. The mechanisms used by IE1 to activate viral gene expression perhaps all depend on IE1-protein interactions. Therefore, it is important to identify the cellular proteins interacting with IE1 at a global level.

## 2. Results

One of the major challenges in the post-genomic era is to explore the functional elements in the human genome. It also applies to the virus-host interaction. Identifying the cellular proteins that interact with the important virus proteins will certainly contribute to the understanding of the mechanisms that the virus uses for its gene expression and replication. Protein arrays constitute a powerful tool for high throughput and multiplexed protein analysis, including protein detection, the investigation of protein interactions with various types of molecules, and the determination of protein functions [28]. Protein array technology is highly sensitive and generates large amounts of data in a single experiment with comparatively low sample consumption; therefore, it is highly economical [28]. In current studies, we used protein array assays to screen cellular proteins that interact with HCMV IE1. Here we report our experimental results.

**Figure 1:**
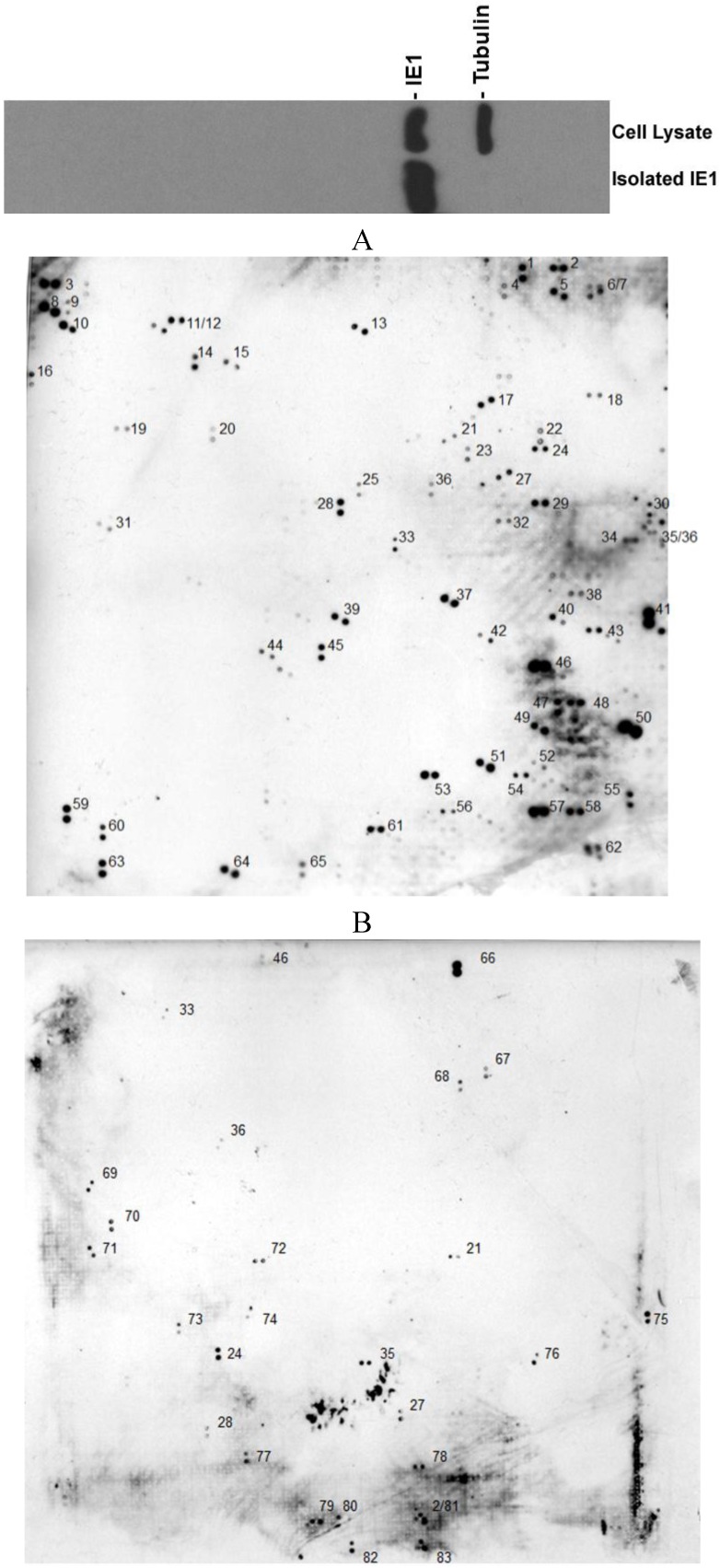
Protein Array. The isolated IE1 was incubated with the two PVDF membranes, which was followed by incubation with a horseradish peroxidase-coupled secondary antibody (GE Healthcare Bio-Sciences, Pittsburgh, PA, USA) and detection with enhanced chemiluminescence (GE Healthcare Bio-Sciences, Pittsburgh, PA, USA), according to standard methods. Some genes overlap between the two PVDF membranes.

First, we isolated the IE1 from an IE1-producing cell line (previously called U373-IE1, now called U-251 MG-IE1) [29] using a specific anti-IE1 antibody that was later incubated with beads-conjugated secondary antibody after which the beads were washed in binding/wash buffer (20 mM Na_2_HPO_4_, 0.15 M NaCl, pH 7.0). The pulled-down IE1 was washed off from the secondary antibody-bound beads with elution buffer (0.1 M glycine, pH 2–3), and the eluted solution was immediately neutralized with neutralization buffer (1 M Tris, pH 8.5). The isolated IE1 was confirmed by Western blot assay as shown in the top of Figure 1. Then the isolated IE1 was incubated at room temperature for 1 hour with the 22 cm × 22 cm PVDF membranes presenting up to 7390 *in situ* expressed human proteins (Cat# Unipex_1, _2, library # 9027, #9028, imaGenes GmbH, Berlin, Germany). After 24 h of incubation with primary anti-IE1 antibody at 4 °C, the membrane was washed with the buffer 2 times followed by incubation with a horseradish peroxidase-coupled secondary antibody (GE Healthcare Bio-Sciences, Pittsburgh, PA, USA) and detection with enhanced chemiluminescence (Pierce, Rockford, IL, USA), according to standard methods. As can be seen in Figure 1A,B, 83 cellular proteins were found to bind to IE1. In the PVDF membrane, each *in situ* expressed cellular protein is presented in doublets, which is why we can see 2 dots together for each protein. We set up cutoffs according to the background index. Figure 1A,B represents 2 PVDF membranes that were processed at the same time with the same samples. The 83 proteins are listed in the Table 1 with their Gene bank #, name, function, and references. 

Further analysis of the 83 proteins revealed some important information: First, some of them have already been reported to interact with IE1. These are ATRX (#13) [15], splicing factor, arginine/serine-rich 2 (#40) [18], and Daxx (#65) [30]. Second, some of them are reported to be important for CMV infection but they have not been shown to interact with IE1, these are CDC25A (#10) [31], ATF4 (#41) [32], BCL-2 (#46) [33], and Ataxin (#39) [34]. In addition, 7 of the proteins that interact with IE1 are RNA binding proteins: RNA binding motif protein 10 (RBM10) (#5), RNA binding motif protein 5 (#20), Staufen, RNA binding protein (#26), splicing factor, arginine/serine-rich 2 (#40), heterogeneous nuclear ribonucleoprotein F (#52), RD RNA binding protein (#59), and PRP40 pre-mRNA processing factor 40 B (#60). All the RNA-bonding proteins are important for pre-mRNA processing. To our surprise, neither HDAC1, 2, or 3 nor TRIM19 (PML) is on the list; however, HDAC10 (#15) and TRIM33 (#57) are both detected as IE1-binding proteins with high signals (Figure 1).

**Figure 2:**
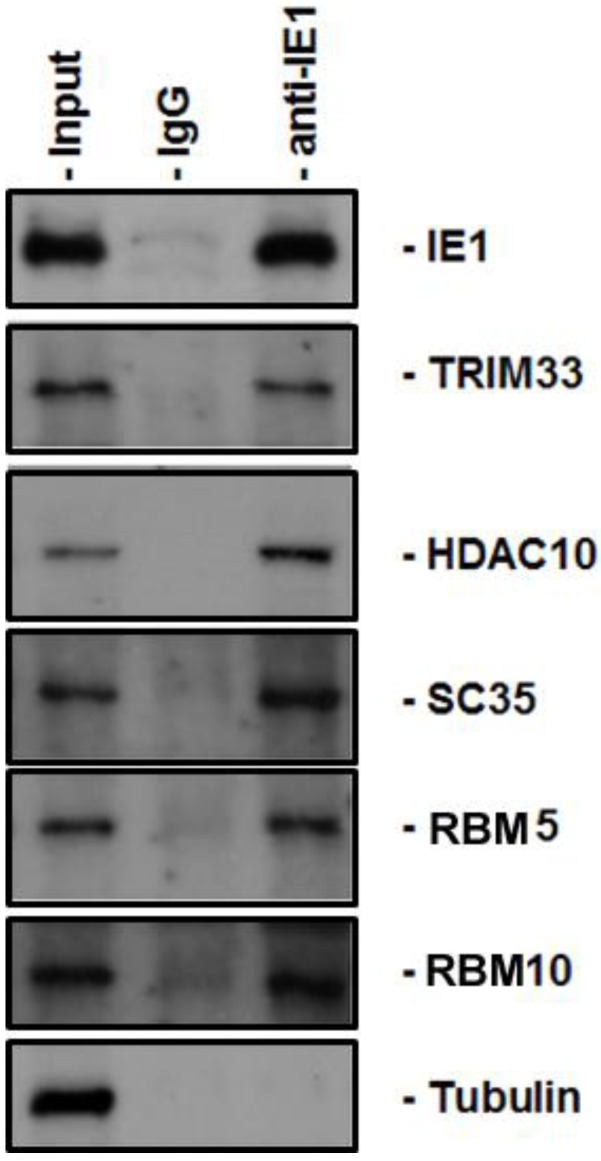
Validation of some IE1 PPIs by coIP. The nuclear extract from the IE1-producing cell line was prepared and incubated with anti-IE1 antibody followed by incubation with secondary bound beads. The pulled-down complexes were examined by Western blot using antibodies against the protein, as indicated on the right hand side.

Since most of the IE1-interacting proteins in the Table 1 have not been reported, we need to validate the reliability of the results from the protein arrays. For that purpose, we performed a coimmunoprecipitation (coIP) assay using antibodies on hand to examine whether the proteins can be pulled down by the anti-IE1 antibody. The IE1-producing cell line (U-251 MG-IE1) [29] was cultured at 37 °C until it reached 95% confluence. The nuclear extract was prepared according to the protocol as described in Material and Method section. The nuclear extracts were incubated with anti-IE1 antibody or with normal IgG and then secondary antibody-conjugated beads. After several washings, the eluted complexes from the beads were examined by Western blot assay using antibodies against the proteins as indicated in Figure 2. All the proteins from the list that are examined by Western blot assay were positive. We also examined the tubulin as control and it was negative. Therefore, the results from the protein arrays are reliable. 

**Table 1:** List of the Human Cytomegalovirus (HCMV) IE1 Protein-Protein Interactions (PPIs) by Protein Array Assay. The positive spots in Figure 1A,B were listed in this table according to the manufacturer’s annotation table. The dots are all in doublets. Some genes overlap between the two PVDF membranes. Cellular Proteins Interacting with Human Cytomegalovirus IE1 (Identified by Protein Array Assays).

GenBank entry#	Name of the protein	Function	Ref.
AF217982	CDK5 regulatory subunit associated protein	Tumorigenesis and metastasis	[35]
D84294	TPRDI (tetratricopeptide repeat)	Down syndrome	[36]
BC069268	Golgi autoantigen, golgin subfamily a	Tentacular matrix	[37]
NM_007144	Polycomb group ring Finger 2 (PCGF2)	Embryogenesis and tumorigenesis	[38]
NM_005676	RNA binding motif protein 10 (RBM10)	RNA-related apoptosis	[39]
Z11584	NuMA protein	Spindle orientation	[40]
BC022880	Breast carcinoma amplified sequence 2	Negative regulator of p53	[41]
BC057387	LSM14B, SCD6 homolog B	Regulation of translation	[42]
BG354577	CDCA4 Cell division cycle associated gene 4	Transcription regulation	[43]
NM_001789	Cell division cycle 25 homolog A (CDC25A)	Cell cycle regulation	[44]
NM_003026	SH3-domain GRB2-like 2 (SH3GL2)	Tumorigenesis	[45]
NM_203505	GTPase activating protein binding protein 2	Stress granule formation	[46]
XM_001128623	Transcriptional regulator ATRX	Transcriptional repression	[47]
NM_153273	Inositol hexaphosphate kinase 1 (IHPK1)	Type 2 diabetes	[48]
NM_032019	Histone deacetylase 10 (HDAC10)	Cancer metastasis	[49]
NM_001040653	ZXD family zinc finger C (ZXDC)	MHC gene transcription	[50]
NM_138383	Actin-bundling protein with BAIAP2 homology (ABBA-1)	unknown	[51]
NM_001082486	Adrenocortical dysplasia homolog (mouse) (ACD), transcript variant 1	Tumorigenesis	[52]
AK122898	ADP-ribosylation factor binding protein GGA1	Trans-olgi network	[53]
AB208813	RNA binding motif protein 5	Gene splicing factor	[39]
NM_170677	Meis homeobox 2 (MEIS2)	Unknown	[54]
NM_000038	Adenomatosis polyposis coli (APC)	Tumor supresor	[55]
NM_003660	Protein tyrosine phosphatase, receptor type f polypeptide (PTPRF), interacting protein (liprin), alpha 3 (PPFIA3)	Unknown	[56]
NM_012398	Phosphatidylinositol-4-phosphate 5-kinase, type I, gamma (PIP5K1C)	Lethal contractural syndrome	[57]
NM_003861	WD repeat domain 22 (WDR22)	Unknown	N/A
NM_017453	Staufen, RNA binding protein	mRNA traffic	[58]
BC017222	Sequestosome 1	Signal transduction	[59]
NM_152586	Ubiquitin specific peptidase 54 (USP54)	Unknown	[60]
NM_014868	Ring finger protein 10 (RNF10)	Type 2 diabetes	[61]
BC041897	SplA/ryanodine receptor domain and SOCS box containing 3	Inflammation	[62]
AB209534	Tumor rejection antigen (gp96) 1	Unknown	N/A
NM_001080424	Jumonji domain containing 3 (JMJD3)	Histone demethylation	[63]
NM_006312	Nuclear receptor co-repressor 2 (NCOR2)	Gene suppressor	[64]
AK124656	Gamma enolase (EC 4.2.1.11)	Neural tissue development	[65]
AK123065	Sperm acrosomal protein	Motility of the spermatozoon	[66]
NM_001098800	Melanoma antigen family D 4 (MAGED4)	Renal cell carcinoma	[67]
NM_021098	Calcium channel, voltage-dependent, T type, alpha 1H subunit (CACNA1H)	T-type Ca(2+) channel activity	[68]
NM_001417	Eukaryotic translation initiation factor 4B (EIF4B)	Translation control	[69]
NM_002973	Ataxin 2 (ATXN2)	Spinocerebellar ataxia type 2	[70]
BC070086	Splicing factor, arginine/serine-rich 2	Gene splicing	[18]
NM_001675	Activating transcription factor 4 (ATF4)	Gene transcription	[71]
AB209149	Phenol-sulfating phenol sulfotransferase 1	Transfer of a sulfonate moiety	[72]
AY509035	Roundabout-like protein 3 (ROBO3)	Horizontal gaze palsy	[73]
NM_014751	Metastasis suppressor 1 (MTSS1)	Metastasis	[74]
AY335491	GON4L isoform C (GON4L)	Hematopoiesis	[75]
NM_014739	BCL2-associated transcription factor 1	Transcriptional repression	[76]
AB067518	KIAA1931 protein	Unknown	N/A
NM_003200	Transcription factor 3 (E2A immunoglobulin enhancer binding factors E12/E47) (TCF3)	Transcription regulation	[77]
AB209197	Protein phosphatase 1 (PP1)	Multiple functions	[78]
NM_020226	PR domain containing 8 (PRDM8)	Gene repressor	[79]
XM_166659	OTU domain containing 1 (OTUD1)	Unknown	N/A
NM_001098208	Heterogeneous nuclear ribonucleoprotein F (HNRPF)	Gene splicing	[80]
AB209441	Fibroblast growth factor receptor 3 isoform 1 precursor	Development	[81]
NM_152643	Kinase non-catalytic C-lobe domain (KIND) containing 1 (KNDC1)	Unknown	N/A
NM_001982	V-erb-b2 erythroblastic leukemia viral oncogene homolog 3 (ERBB3)	Cell proliferation or differentiation	[82]
NM_015695	Bromodomain and PHD finger containing, 3 (BRPF3)	Fetal liver erythropoiesis	[83]
NM_015906	Tripartite motif-containing 33 (TRIM33)	Tumor suppressors	[84]
NM_032127	Chromosome 11 open reading frame 56 (C11orf56)	FTS and Hook-interacting protein	[85]
NM_002904	RD RNA binding protein (RDBP)	Repress RNA polymerase II	N/A
NM_012272	PRP40 pre-mRNA processing factor 40 B (PRPF40B)	Gene splicing	[86]
NM_020967	Nuclear receptor coactivator 5 (NCOA5)	Gene regulator	[87]
NM_001080495	KIAA1856 protein (KIAA1856)	Unknown	N/A
NM_022748	Tensin 3 (TNS3)	Signal transduction	[88]
BC063642	Phosphodiesterase 4D interacting protein (myomegalin)	Control microtubules	[89]
AB209493	Death-associated protein 6 (DAXX)	Development and Cancer	[90]
NM_003482	Myeloid/lymphoid or mixed-lineage leukemia 2 (MLL2)	Lymphomagenesis	[91]
NM_033396	Tankyrase 1 binding protein 1, (TNKS1BP1)	Unknown	[92]
AY729650	Intersex-like protein	Unknown	[93]
BC110647	Immediate-early response 2	Unknown	N/A
AB014581	KIAA0681 protein	Unknown	N/A
NM_004235	Kruppel-like factor 4 (gut) (KLF4)	Transactivation and growth suppression	[94]
AB051455	KIAA1668 protein	Unknown	N/A
AF045458	Serine/threonine kinase ULK1 (ULK1)	Autophagy activation	[95]
NM_001003694	Bromodomain and PHD finger containing, 1 (BRPF1)	Unknown	N/A
NM_003626	protein tyrosine phosphatase, receptor type, f polypeptide (PTPRF), interacting protein (liprin), alpha 1 (PPFIA1)	Unknown	N/A
NM_006291	tumor necrosis factor, alpha-induced protein 2 (TNFAIP2)	Unknown	N/A
AB209643	smoothelin isoform b	Unknown	N/A
NM_002857	peroxisomal biogenesis factor 19 (PEX19)	Peroxisomal assembly	[96]
AB061669	receptor for advanced glycation end-products	Signaling and inflammation	[97]
NM_014678	SAPS domain family, member 2 (SAPS2)	Unknown	N/A
NM_006887	zinc finger protein 36, C3H type-like 2 (ZFP36L2)	Unknown	N/A
AB208876	axin 1 isoform	Wnt signaling pathway	[98]
NM_004530	matrix metallopeptidase 2 (MMP2)	Metastasis and inflammation	[99]

## 3. Discussion

HCMV infects a large part of the population and has serious consequences for immunocompromised persons with AIDS or who have received organ transplants and for newborns after congenital infection. In fact, HCMV is the leading viral cause of congenital birth defects [1]. More than 30% of primary HCMV infections in pregnant women result in placental transmission and clinical syndromes. Congenital infection is not uncommon [100,101,102]. Of symptomatic newborns, about 12% die and half of the survivors develop mental retardation, vision loss, and/or sensorineural deafness [103]. Interference strategies commonly target the early events of the replication cycle by using approved nucleoside analogs such as ganciclovir, the nucleotide analog cidofovir, and foscarnet. However, these can lead to resistance [104]. *In vitro* anti-sense oligonucleotides against the HCMV immediate-early protein 2 (IE2) have proven effective [105], as has targeting the UL36 and UL37 sites [105,106]. These attempts show that targeting the HCMV IE part of the propagation cycle may be effective; however, none of these treatments are permissible or feasible for use on a potentially infected fetus. The lack of suitable treatment modalities has especially serious consequences for the congenitally infected fetus and for patients with impaired immune systems. The challenge is to find treatment modalities that do not depend on the inhibition of the DNA replication process. This has attracted our investigation to the immediate-early (IE) stage of HCMV infection. Understanding IE1’s function is our first step in the development of a practical strategy against HCMV replication via interfering with IE1 function.

In many cases, proteins play their biological role through interactions with other proteins. Especially for viral infection, viruses need to manipulate cellular function via interacting with cellular proteins. Therefore, the systematic identification and characterization of protein-protein interactions (PPIs) is considered a key strategy to understand protein function. In the case of HCMV viral immediate-early products, IE1 might be the most important protein that initiates the early interactions between the virus and cells. The number of possible contacts between protein surfaces is astronomical. In this context, protein array technology opens up new avenues for the characterization of viral proteins and the identification of molecular partners involved in virus-mediated regulatory networks in cells. The array technology used in the present study started with incubation of IE1 with the PVDF membranes that carry GST-tagged human fusion proteins. Those fusion proteins were expressed in *E. coli*, and after native lysis with lysozyme, crude protein extracts were prepared under non-denaturing conditions in a 384-well plate format. The crude bacterial cell extracts were used for incubation overnight with the high-density spotted PVDF membranes, which were used to detect IE1-interacting proteins. 

Using the protein array, eighty-three cellular proteins in total were identified to be PPIs of HCMV IE1. Most of them were gene regulators. Only 3 IE1 PPIs from the list have been reported. However, many HCMV-mediated cell biological activities have been linked to IE1 (which link is also shown in these array assays); these activities include, among others, the interaction of IE1 with BCL-2, CDC25A, which interaction might be important for HCMV-induced “G1 arrest”. Some cellular proteins reported to interact with IE1 are not on the list, they might interact with IE1 only indirectly or the printed proteins are in different isoforms. For example, some isoforms of PML was found to interact with IE1 [15] but is not on the list because the other isoforms of PML are printed on the PVDF membranes. As shown on the Table 1, 7 RNA binding proteins are found to interact with IE1, implying that IE1 has a novel function in viral mRNA processing. 

In conclusion, our current effort in characterizing IE1’s PPIs resulted in the identification of 83 cellular proteins that directly interact with HCMV IE1. The results have been partially validated by coIP assay. With the data listed in the Table 1, we will further investigate the function of the PPIs of IE1.

## 4. Experimental

### 4.1. Tissue Culture and Viruses

The IE1-producing cell lines (U-251 MG-IE1) [29] were maintained in Dulbecco’s modified Eagle’s medium (DMEM) supplemented with 10% fetal calf serum (FCS) and 1% penicillin-streptomycin. 

### 4.2. Antibodies

The monoclonal antibody against IE1 (MAB810) and the rabbit antibodies against cellular proteins were purchased from Santa Cruz Biotechnology, Inc. (Santa Cruz, CA, USA). The rabbit antibodies included anti-SC35 (sc-28720), -TRIM33 (sc-68424), -HDAC10 (sc-130775), and -tubulin (sc-5546). Rabbit antibodies against RBM5 (ab69970) and RBM 10 (ab126112) were bought from Abcam (Cambridge, MA, USA)

### 4.3. Preparation of Nuclear Extracts

Nuclear extracts were obtained essentially as described previously [15]. Briefly, monolayer cells were washed with PBS and scraped into fresh Eppendorf tubes. Cell pellets were resuspended in cold buffer A (10 mM HEPES-KOH, pH 7.9, at 4 °C, 1.5 mM MgCl_2_, 10 mM KCl, 0.5 mM dithiothreitol, 0.2 mM phenylmethylsulfonyl fluoride) and incubated for 10 min at 4 °C. Then the cells were dounced with 10–20 plunges in a Kontes-B (Wheaton, DC, USA) Dounce Homogenizer (Pestle B) and poured into new bottles after centrifugation. The pellets were resuspended in cold buffer C (20 mM HEPES-KOH, pH 7.9, 25% glycerol, 420 mM NaCl, 1.5 mM MgCl_2_, 0.2 mM EDTA, 0.5 mM dithiothreitol, 0.2 mM phenylmethylsulfonyl fluoride) by vortexing and incubated for 30 min at 4 °C. The pellets were dounced again with 10–20 plunges in Kontes-B (Wheaton) Dounce Homogenizer (Pestle B), and clarified extracts were transferred to fresh tubes and stored at −70 °C until use. 

### 4.4. Coimmunoprecipitation

Antibodies were coupled to protein G-Sepharose beads (GE Healthcare Bio-Sciences, Pittsburgh, PA, USA), according to the manufacturer’s instructions. After a wash with PBS-0.1% bovine serum albumin, the beads were incubated overnight at 4 °C with clarified extracts, washed again in PBS-0.1% bovine serum albumin, and resuspended in a mixture of PBS and 2× Laemmli buffer (20 µL of each). After being heated for 5 min at 95 °C, the beads were removed by centrifugation and supernatants were analyzed by SDS-PAGE and immunoblotting.

### 4.5. Immunoblot Analysis

Proteins were separated by sodium dodecyl sulfate-7.5% polyacrylamide gel electrophoresis [107] (10 to 20 µg loaded in each lane), transferred to nitrocellulose membranes (Amersham Inc., Piscataway, NJ, USA), and blocked with 5% nonfat milk for 60 min at room temperature. Membranes were incubated overnight at 4 °C with primary antibody followed by incubation with a horseradish peroxidase-coupled secondary antibody (Amersham Inc.) and detection with enhanced chemiluminescence (Pierce, Rockford, IL, USA), according to standard methods. Membranes were stripped with stripping buffer (100 mM β-mercaptoethanol, 2% SDS, 62.5 mM Tris-HCl, pH 6.8), washed with PBS-0.1% Tween 20, and used to detect additional proteins.

## 5. Conclusions

We currently used a protein array assay to identify HCMV IE1 interacting proteins. There are 83 cellular proteins that are PPIs of IE1 and most of the PPIs have not been reported previously. The interactions have been partially validated by coIP method which confirmed that the protein array assay is reliable. 
